# Effect of palliative care decisions making on hospital service use at end-of-life in patients with malignant brain tumors: a retrospective study

**DOI:** 10.1186/s12904-023-01154-z

**Published:** 2023-04-10

**Authors:** Nelli-Sofia Nåhls, Riikka-Leena Leskelä, Tiina Saarto, Outi Hirvonen, Anu Anttonen

**Affiliations:** 1 Department of Oncology, Vaasa Central Hospital, Vaasa, Finland; 2 Nordic Healthcare group, Helsinki, Finland; 3 Department of Palliative Care, Comprehensive Cancer Center, Faculty of Medicine, Helsinki University Hospital, Helsinki University, Helsinki, Finland; 4 Palliative Center, Turku University Hospital, Turku, Finland; 5 Department of Clinical Oncology, University of Turku, Turku, Finland; 6 Department of Radiotherapy, Comprehensive Cancer Center, Helsinki University Hospital, Helsinki, Finland; 7 Department of Oncology, Comprehensive Cancer Center, Helsinki University Central Hospital, Helsinki, Finland

**Keywords:** Glioblastoma, Palliative care decision, End of life, Emergency department, Hospital services

## Abstract

**Background:**

Palliative care (PC) improves Quality of life and reduces the symptom burden. Aggressive treatments at end of life (EOL) postpone PC. The aim of this single-center retrospective study was to evaluate the timing of the PC decision i.e., termination of cancer-specific treatments and focusing on symptom-centered PC, and its impact on the use of tertiary hospital services at the EOL.

**Methods:**

A retrospective cohort study on brain tumor patients, who were treated at the Comprehensive Cancer Center of the Helsinki University Hospital from November 1993 to December 2014 and died between January 2013 and December 2014, were retrospectively reviewed. The analysis comprised 121 patients (76 glioblastoma multiforme, 74 males; mean age 62 years; range 26–89). The decision for PC, emergency department (ED) visits and hospitalizations were collected from hospital records.

**Results:**

The PC decision was made for 78% of the patients. The median survival after diagnosis was 16 months (13 months patients with glioblastoma), and after the PC decision, it was 44 days (range 1-293). 31% of the patients received anticancer treatments within 30 days and 17% within the last 14 day before death. 22% of the patients visited an ED, and 17% were hospitalized during the last 30 days of life. Of the patients who had a PC decision made more than 30 days prior to death, only 4% visited an ED or were hospitalized in a tertiary hospital in the last 30 days of life compared to patients with a late (< 30 days prior to death) or no PC decision (25 patients, 36%).

**Conclusions:**

Every third patient with malignant brain tumors had anticancer treatments during the last month of life with a significant number of ED visits and hospitalizations. Postponing the PC decision to the last month of life increases the risk of tertiary hospital resource use at EOL.

## Introduction

High grade gliomas are the most common primary brain tumor, affecting the patients’ lifespan and causing significant suffering especially at the end of the life (EOL) [1–3]. Aggressive treatments of glioblastoma multiforme (GBM), including surgery, radiotherapy or chemoradiotherapy and adjuvant chemotherapy, have not been able to improve median survival over 15 months. The 5-year survival rate is less than 10%. [3–7]

Brain tumor patients, especially GBM patients, suffer from severe neurological symptoms caused by the tumor itself or side effects of treatment, e.g., seizures, headaches, drowsiness, and neurological defects, throughout the disease trajectory. Early cognitive and behavioral decline are also common in patients diagnosed with GBM [3, 8–10]. Previous studies have shown that many patients with GBM end up in an emergency department (ED) with these symptoms [11, 12]. According to a single-institution retrospective study, the majority of patients with GBM (71%) utilized acute care, 42% had multiple visits to an ED and 38% had an ED visit within 30 days of death. The most common reason for visiting an ED was seizure [11].

It has been shown that early integration of palliative care (PC) enhances cancer patients’ quality of life and decreases the use of aggressive treatments at the EOL, ED visits and hospitalizations [10, 13–20].

A previous Finnish study for heterogeneous group of cancer patients showed that continuation of anticancer treatments until the last months of life increased ED visits and hospitalizations at tertiary hospital. On the contrary, contact to PC unit together with earlier interruption of anticancer treatments reduced the use of tertiary hospital resources [20].

Finnish brain tumor patients are treated mainly at public university and central hospitals. Department of Oncology at Helsinki University Central Hospital (HUCH) is the largest university hospital in Finland being responsible for the cancer care of a population of approximately 1.6 million in Southern Finland.

To our knowledge, there are no studies of the timing of PC decisions and their influence on resource use for malignant glioma patients at the EOL. The aim of our retrospective cohort study was to investigate the timing of a decision to terminate oncological treatments (except palliative radiotherapy) and focus on symptom-centered PC and its impact on the use of hospital services. The secondary aim was to investigate the care pathway of brain tumor patients from diagnosis to EOL care.

## Patients and methods

### Cohort selection

One hundred and twenty-four brain tumor patients were identified from hospital registries. Inclusion criteria’s were the International Classification of Disease (ICD-10) coding for neoplasma malignum cerebri (C71.1-71.9), including grade II-IV gliomas, and visit at the Comprehensive Cancer Center of the Helsinki University Hospital between 1993 and 2014 and died between 2013 and 2014. The only exclusion criteria was pediatric (< 18-year old) patients. One hundred and twenty-one patients fulfilled the inclusion criteria as three pediatric patients were excluded.

### Data collection

Data on patient demographics (age, sex) and tumor (cancer diagnosis) characteristics, cancer treatments (chemotherapy and radiotherapy), data on visits to the Palliative Care Center, Psychosocial Support Unit and ED, and hospitalizations at tertiary hospital were retrospectively collected from the hospital medical records. In addition, the time point of do not resuscitate (DNR) orders, PC decisions, reasons to visit the ED and referrals to EOL care were collected manually from the hospital charts. Data on the cause, date and place of death were collected from death certificates [21]. The coordinating ethical committee of Helsinki and Uusimaa district have approved the study protocol (258/13/03/00/15).

This study does not contain information about the number of ED visits or inpatient days in primary care services because this information is not available in the hospital medical records.

### Palliative care decision and period

The PC decision is defined as a decision to terminate curative or life-prolonging anticancer treatments and focus on symptom-centered PC, and the PC period as a period of disease where curative or life-prolonging treatment can no longer be offered. The PC period covers the period from the decision to abstain from cancer-specific treatment (except palliative radiotherapy) to death. The PC decision was defined as an early decision, if it was made 30 days before death and late, if the PC decision was given during the last 30 days before death.

### Statistical analyses

Descriptive statistics are reported as medians and ranges, numbers of incidences and percentages. Comparisons between categorical variables were performed using the χ2 test (CHISQ. TEST.RT) in Microsoft Excel 201 version 2108. For the statistical testing categories for place of death had to be grouped into two groups (“specialized” and “general”) in order to obtain the expected size of > 5 for each group. Overall survival was calculated from the date of pathologic diagnosis to the date of death. The survival after the PC decision was calculated from the date of the PC decision to the date of death.

## Results

Patient characteristics are shown in Table 1. The final cohort comprised 121 patients, 76% of whom were diagnosed with GBM. The median age was 62 years; 61% of the patients were male. Most of the patients received primary treatment with curative or life prolonging intent. PC was the primary treatment for 13% of the patients, 51% of the patients received chemotherapy for progression. Temozolomide, lomustin and bevacizumab alone or with lomustin were most commonly used.

52% of the patients received palliative radiotherapy (10 as primary treatment and 53 as secondary treatment). The most commonly used dose was 30 Gy in 3 Gy daily fractions. The majority of the patients (97%) completed the radiotherapy.

**Table 1:** Patient characteristics

Patients N=121	Number (%)
Age in years median (range) ≤65 years >65 years	62 (26-89) 77 (64%) 44 (36%)
Sex	
Male Female	74 (61%) 47 (39%)
Primary diagnosis	
Glioblastoma Astrocytoma Oligodendroglioma Oligoastrocytoma Others or no histological diagnosis	92 (76%) 10 (8%) 5 (4%) 8 (7%) 6 (5%)
Surgical interventions	
Operation Biopsy No operation or biopsy	108 (89%) 11 (9%) 2 (2%)
Primary treatment with curative or life prolonging intent	
Chemoradiotherapy and adjuvant chemotherapy Chemoradiotherapy Radiotherapy and adjuvant chemotherapy Radiotherapy Chemotherapy Palliative radiotherapy Supportive care only	62 (51%) 17 (14%) 3 (2%) 21 (17%) 2 (2%) 10 (8%) 6 (5%)
Systemic anticancer treatments after primary treatment	
1st line 2nd line 3rd line or more	28 (23%) 20 (17%) 14 (12%)
Re-irradiation courses after primary treatment	
One Two Three or more	43 (36%) 9 (7%) 1 (1%)
Median survival (time and range)	
After diagnosis Glioblastoma Other histology After PC decision Glioblastoma Other histology	16 months (1-238) 13 months (1-95) 51 months (2-238) 44 days (1-293) 38 days (0-270) 71 days (1-293)

### Care during the last 30 days of life

31% of the patients received anticancer treatments during the last 30 days of life (Table 2). 21% of the patients received systemic anticancer treatment, and 10% of the patients received chemoradiotherapy or radiotherapy.

22% of the patients visited an ED in the last 30 days of life, and three-quarters (21 patients) of those patients were admitted to a tertiary hospital. The average length of stay in the hospital was 8 days (a range of 2–16 days). 52% of the patients died during the hospital stay. None of the patients were transferred to an intensive care unit. (Table 2) The most common reasons for visiting the ED 30 days before death were neurological symptoms (48%) such as mental confusion, seizure, palsy, and deterioration in the patient’s overall clinical health status (24%). There were also ED visits for infection (14%).

**Table 2:** Service use and timing of palliative care decisions

	Number (%)
Last anticancer treatment	
60 days before death 30 days before death 14 days before death	52 (43%) 37 (31%) 20 (17%)
“Do not resuscitate” order Made by a clinical oncologist Made by another specialist	46 (38%) 17 (37%) 29 (63%)
Number of patients who visited the Palliative Care Center Median number of visits per patient (range)	22 (18%) 1 (1–6)
Number of the patients who visited the Psychosocial Support Unit Median number of visits per patient (range)	24 (20%) 2 (1–12)
PC decision	94 (78%)
Timing	
> 30 days before death ≤ 30 days before death	51 (42%) 43 (36%)
Number of the patients who used tertiary hospital services in the last 30 days of life	
Emergency department visits Hospitalization at university hospital	27 (22%) 21 (17%)
Place of death	
Home Hospice Specialized palliative care ward in a community hospital General primary care hospital ward Tertiary hospital Nursing home	7 (6%) 24 (20%) 24 (20%) 51 (42%) 10 (8%) 5 (4%)

### Palliative care decision and visit to palliative care unit

The effect of the timing of the PC decision on the use of hospital services is presented in Table 3. The PC decision was made for 78% of the patients. The median survival after the PC decision was 44 days (range 1-293). (Table 2). Age, diagnosis (GBM vs. other) and the number of treatment lines given did not correlate with the timing of the PC decision. However, the longer the overall survival, the more likely the PC decision was made earlier than 30 days before death (χ2 = 7,1, p = 0,03).

Of the patients who had a PC decision made more than 30 days prior to death, only 4% of patients visited an ED or were admitted to a tertiary hospital in the last 30 days of life, whereas of the patients with no PC decision or a PC decision made within 30 days of death, the number was 25 (36%). (Table 3)

Only about a fifth of the patients visited the Palliative Care Center (18%) and Psychosocial Support Unit (22%). All the patients (N = 22) who visited the Palliative Care Center, except for one patient, had a decision for PC. The median survival after first contact with the Palliative Care Center was 100 days (a range of 8-310).

40% of the patients died at specialized PC services. EOL care at home was rare. There was no significant association between timing of the PC decision and place of death (Table 3).

**Table 3:** The effect of PC decision timing on the use of hospital services

Number of patients N = 94	Late PC decision (≤ 30 days) (N = 43)	Early PC decision (> 30 days) (N = 51)	P-value (χ2 test)
Last anticancer treatment			< 0.001
No treatment 60 days before death 60 − 31 days before death 30 − 14 days before death < 14 days before death	15 (16%) 2 (2%) 14 (15%) 12 (13%)	41 (44%) 8 (9%) 0 2 (2%)	
“Do not resuscitate” order Made by a clinical oncologist Made by another specialist	19 (20%) 7 (37%) 12 (63%)	18 (19%) 10 (56%) 8 (44%)	0.379
Number of the patients who visited the Palliative Care Center	4 (4%)	17 (18%)	0.005*
Number of the patients who visited the Psychosocial Support Unit	9 (10%)	9 (10%)	0.687
Number of the patients who used tertiary hospital services in the last 30 days of life			
Emergency department visits Hospitalization at university hospital	18 (19%) 15 (16%)	2 (2%) 0 (0%)	< 0.001* < 0.001*
Place of death			0.336
Home Hospice Specialized palliative care ward in a community hospital General primary care hospital ward Tertiary hospital Nursing home	1 (1%) 8 (9%) 8 (9%) 20 (21%) 5 (5%) 1 (1%)	4 (4%) 14 (15%) 10 (11%) 19 (20%) 1 (1%) 3 (3%)	

*significant at 0.01 level

## Discussion

The real-life data of the brain tumor patient care pathway demonstrate aggressive EOL treatment and poor integration into PC services. Late or no PC decisions, e.g., the decision to terminate curative or life-prolonging anticancer treatments and focus on symptom-centered PC, increased the need for acute services.

Our study population was a deceased brain tumor population treated in a university hospital. In our patient population, 76% had GBM. Overall survival in the present study was relatively short (16 months in all brain tumor patients and 13 months patients with GBM), but in line with the previous studies. Previous retrospective studies [5, 6] have reported similarly short survival rates. In a study of 2,045 GBM patients, Korja et al. [6] reported a median survival of 11.7 months in patients under 70 years old. With patients over 75 years old, the median survival was only 4.5 months. Stupp et al. [5] reported a median survival of 12.1 months with GBM patients who only received radiotherapy and 14.6 months with patients who received combined treatment (chemoradiotherapy and adjuvant temozolomide).

In our study, anticancer treatments (first-line radiotherapy or chemoradiotherapy) were in line with the international guidelines [4–22]. On the other hand, our patients received slightly more oncological treatments than those in the literature. The majority of our patients (85%) underwent tumor resection or biopsy and received adjuvant treatment. Only 5% of the patients did not receive any treatment. For comparison, in a large retrospective cohort study of 16,717 patients with newly diagnosed GBM, 60% of patients received radiotherapy and chemotherapy or radiotherapy alone, and 33% received no therapy [23]. In another study of 2,836 GBM patients over 70 years of age, 46% of patients underwent surgery and received radiotherapy. 14% of the patients did not undergo either treatment [24]. This difference can be at least partly explained by the lower rate of GBM patients compared to previous studies. Another plausible explanation could be patient selection as in our study only patients who were treated in university hospital were included compared to population based large cohorts.

The same trend was seen in the treatment at progression. One-fourth (25%) of our patients were not treated with second-line therapy at progression, which is somewhat less than in previous reports. A retrospective multicenter analysis of 299 patients with recurrent GBM after first-line treatment reported that 39.5% of the patients did not receive any oncological treatment at progression [25]. Shi et al. [26] reported that 267 out of 637 (42%) patients who progressed after primary treatment for newly diagnosed GBM received neither reirradiation nor systemic treatment at progression. Furthermore, a significant number of the patients in our study (31%) continued oncological treatments during the last 30 days of life. Contrary, in the study of Kuchinad et al. [3], 17 out of 100 GBM patients were treated with chemotherapy in the last 4 weeks of life. This is significantly higher than what we have previously reported from a heterogeneous cancer population of the same region. In all cancer patients, the corresponding figure was 10% [27]. Thus, it seems that the treatment policy was somewhat more aggressive in the present cohort. Interestingly, however, in our cohort the timing of PC decision has a significant impact on aggressiveness of the treatment at end-of-life. Early made PC decision (> 30 days before death) reduced aggressive treatments compared to late decision making. There were similar findings in Harrison’s et al. [28] study, which showed that timing of referral to PC also has an effect on the aggressiveness of the treatment although the PC referral itself did not affect.

PC decision was defined for the majority of the patients (78%) but rather late (median 44 days before death). Very few patients were referred to a PC unit (18%), and this occurred only after the decision to terminate cancer-specific treatment was made, as early integrated PC practice was not used during the study time. Our finding is in line with previous findings that early involvement of PC services is rare in neuro-oncology [20, 29]. One retrospective analysis of 117 deceased GBM patients found that only a third of patients (37%) received PC consultation [13], even though studies have shown that early integrated PC improves quality of life and reduces the use of aggressive treatments at the EOL without impairing survival [30, 31].

Our study indicates that early PC decisions prevent ED visits at the EOL. More than 90% of the patients who visited an ED and were hospitalized in the last 30 days of life did not have a PC decision or it was made very late. Most of those patients who visited an ED (74%) were hospitalized at a university hospital with an average length of stay of 8 days (range 2–16 days). Approximately every second patient (45%) who was hospitalized died during hospitalization. Thus, it seems that an ED visit at the EOL indicates an increased risk of death at a tertiary hospital. Similar findings were found in a single-institution retrospective cohort study, where 37% of GBM patients were hospitalized in the last 4 weeks of life [3]. In addition, Wasilewski et al. [11] reported that 38% of GBM patients had acute care visits within 30 days of death.

In line with our findings, neurological symptoms, most commonly seizures are by far the most common symptom leading to ED and hospitalization among brain tumor patients [11]. More than 50% of patients with GBM experience tumor-related epilepsy [32]. In our study, the most common reasons to visit the ED were neurological symptoms such as mental confusion, seizure and palsy (31%). The next most common reason was deterioration in the patient’s overall clinical health status (24%). Thus, early PC interventions could be particularly valuable in reducing on the use of hospital services at EOL. Investing in PC can have a positive impact on decreasing ED visits and improving documentation on advance care plans [20, 33].

There were some limitations in this study. Our population was quite small and included only patients who had been treated at the Comprehensive Cancer Center of the Helsinki University Hospital. The results do not contain the number of ED visits or inpatient days in primary care services because this information is not in our database. The age of data is another limitation, taking into account the progress that have happened in PC in recent years. At the time of the study, early integrated PC practice was not systematically organized, and it is still not widely available in many countries. Therefore, we believe it is important to show that also timing of PC decision can have an impact on the use of hospital resources. The strength of this study is that we wanted to examine the burden on tertiary hospitals caused by brain tumor patients receiving oncological treatments at EOL. Another strength is the long follow-up period from diagnosis until death.

## Conclusions

Our results are consistent with the literature illustrating that early involvement of PC services is rare in neuro-oncology. Early PC decisions to withhold anticancer treatments and focus on symptom-centered PC reduces the use of tertiary hospital services at EOL. Earlier collaboration between oncologists and PC professionals is needed to improve EOL care for brain tumor patients.

## Data Availability

The data generated during the current study are not publicly available as the data is a part of the larger dataset owned by Helsinki University Hospital. Data are however available from the principal author TS upon reasonable request and with permission of Helsinki University Hospital.

## Abbreviations

PC: Palliative care
EOL: End of life
GBM: Glioblastoma multiforme
ED: Emergency Department
DNR: Do not resuscitate
HUCH: Helsinki University Central Hospital
